# Supplementary material for: A Likelihood Approach for Real-Time Calibration of Stochastic Compartmental Epidemic Models
Source: PLoS Comput Biol. 2017 Jan 17;13(1):e1005257. doi: 10.1371/journal.pcbi.1005257 (PMC5240920; doi:10.1371/journal.pcbi.1005257)
Supplement: S1 File — (TAR.GZ) [file pcbi.1005257.s014.tar.gz › HSPH_Online-SI-Revision/EnKF07/n1-severe/n1-severe_PF_EnKF-summery.pdf]

peak -8	R0	$1/\Theta_2$	inf	inflog	newly	newlylog	Reff	AttackRate
mean	61.7	56.2	1643.3	0.9	7351.	1.1	56.6	140.
median	61.9	54.1	1100.8	0.9	1859.5	1.1	57.7	140.1
Quantile .1	49.3	37.6	107.9	0.3	390.3	0.4	38.8	74.6
Quantile .9	77.9	66.6	3882.7	1.5	5888.3	1.6	74.2	205.8
37	30	12						
length	50							

peak -4	R0	$1/\Theta_2$	inf	inflog	newly	newlylog	Reff	AttackRate
mean	66.4	55.8	233.4	0.6	309.4	0.5	65.9	177.5
median	65.6	54.5	130.4	0.5	245.	0.5	66.6	165.3
Quantile .1	48.5	41.2	81.4	0.3	105.	0.4	59.2	140.9
Quantile .9	85.7	70.9	464.6	0.8	553.6	0.8	72.4	232.5
26	24	0						
length	50							

peak	R0	$1/\Theta_2$	inf	inflog	newly	newlylog	Reff	AttackRate
mean	68.9	57.7	93.9	0.6	147.5	0.5	62.5	186.9
median	68.6	55.5	92.5	0.6	138.6	0.5	63.	186.7
Quantile .1	45.	44.8	78.9	0.4	95.9	0.4	57.3	156.2
Quantile .9	89.	72.	112.6	0.7	185.5	0.6	67.	220.2
20	25	0						
length	50							

peak +4	R0	$1/\Theta_2$	inf	inflog	newly	newlylog	Reff	AttackRate
mean	70.9	53.2	121.8	0.5	204.3	0.5	59.5	177.6
median	69.5	52.7	101.4	0.4	176.3	0.4	59.	172.5
Quantile .1	55.1	34.	68.2	0.3	96.7	0.4	51.3	142.
Quantile .9	87.9	75.4	151.6	0.7	344.5	0.6	69.8	211.7
20	21	0						
length	50							

peak +8	R0	$1/\Theta_2$	inf	inflog	newly	newlylog	Reff	AttackRate
mean	73.1	58.7	4540.7	0.7	5157.9	0.7	57.8	170.7
median	70.8	57.4	152.	0.4	404.	0.5	57.	170.3
Quantile .1	57.4	36.2	87.5	0.3	122.9	0.4	49.4	135.2
Quantile .9	92.	78.3	1035.2	0.9	3141.7	1.1	67.6	206.9
21	28	0						
length	50							
